# Functional diversity of accessory factors of the type VI secretion system

**DOI:** 10.1128/jb.00211-26

**Published:** 2026-08-19

**Authors:** Casey Latario, Benjamin D. Ross

**Affiliations:** 1 Department of Microbiology and Immunology, Geisel School of Medicine, Dartmouth College 3728, Hanover, New Hampshire, USA; University of Virginia School of Medicine, Charlottesville, Virginia, USA

**Keywords:** type VI secretion system

## Abstract

The type VI secretion system (T6SS) is a nanomachine utilized by Gram-negative bacteria to secrete toxic effectors. The T6SS is employed against both prokaryotic and eukaryotic targets, allowing for competitive advantage and virulence of the attackers in a wide variety of environments. Recent work has revealed the great diversity of T6SS systems. Beyond the canonical, conserved architectural genes of the T6SS, diverse T6SS-associated genes (TAGs) are employed to optimize T6SS activity, compensate for variations in architecture, and dictate T6SS use. Although various TAGs have been discovered in T6SS gene clusters, they display unique activities in modifying their cognate T6SS. In this review, we discuss several of the better-studied TAGs, as well as highlight under-researched TAGs that are likely of great significance. As a means to better understand TAG roles, identify common principles and unifying themes, we present the following six functional categories: Firing Coordinators, Target Recognition Factors, Structural Factors, Transcriptional Regulators, Secretion Regulators, and Unknown TAGs.

## INTRODUCTION

Bacteria densely colonize disparate environments, including soil, water, and multicellular hosts. Competition for nutrients and physical space in these environments can be extremely high as genetically diverse microbes interact (1). Bacteria that evolve methods of out-competing their neighbors achieve greater success in dispersal, colonization, and survival in a given environment (2, 3). A major contributor to intraspecies competition is the type VI secretion system (T6SS) (4, 5). Found in ~27% of sequenced Gram-negative bacteria, the T6SS is a contact-dependent contractile injection system which secretes toxic effectors into adjacent competitor cells (6–9). Across this diverse spectrum of bacteria, a myriad repertoire of type VI associated genes (TAGs) can be found adjacent to genes encoding well-conserved structural factors. Each TAG has evolved to facilitate its cognate T6SS role, including interbacterial competition or pathogenesis within a host (10).

Though distributed across diverse bacteria, the T6SS retains a core conserved architecture of 13 proteins (11). These structural components can be classified into three major subcomplexes: the membrane complex (MC), baseplate (BP), and tail-tube complex (TTC). Although exceptions are increasingly being discovered, the T6SS structural components are generally considered to be encoded in a single locus, which also contains the genes encoding the effector and immunity proteins (12–14). Frequently, additional “auxiliary” clusters are present in the bacterial genome carrying effector/immunity gene pairs and TTC components (13, 15).

During T6SS assembly, the bell-shaped MC forms first, constituting a continuous, fivefold symmetric channel from the cytoplasmic side of the inner membrane to the extracellular milieu (16–18). The proteins of the MC vary across phyla, with most Pseudomonadota encoding an MC composed of the proteins TssJLM, while Bacteroidota, for example, have an MC composed of TssNOPQR (16, 18–21). The conserved BP components, TssEFGK, then dock to the cytoplasmically protruding portion of the MC (19, 22). While docking, the BP proteins assemble in hexameric wedges around the trimeric central spike protein, Val-Gly Repeat Protein (VgrG, or “TssI”), which can be paired with Pro-Ala-Ala-Arg Repeat (PAAR) motif proteins to further sharpen the spike (17, 23–25). The BP then serves as an assembly hub and sorting platform for the TTC, including the toxin effectors (23, 26). TTC assembly begins with the initial docking of ring-like, hexameric tail proteins, Hemolysin-Coregulated Protein (Hcp, or “TssD”), to the BP and VgrG (7, 25). As tail subunits polymerize, they become encased in a homodimeric sheath (TssB and TssC), and the effectors are loaded onto VgrG, PAAR, or Hcp (7, 19, 23, 27–29). Though not necessary for all T6SS subtypes, TssA is often considered the final core protein. TssA ensures proper TTC assembly through interactions with several accessory proteins, discussed in detail below (Box 1) (30–33).

Box 1. **TssA**

A TAG that deserves particular emphasis due to the large body of work surrounding it is TssA. TssA is an important coordinator of the TTC in many T6SS*^i^*. Regrettably, its diversity across bacterial systems has led to some confusion in the literature regarding how best to classify TssA. Historically, TssA has been considered one of the 13 core T6SS genes, likely a BP component alongside TssEFG (30). This categorization was prompted by findings showing TssA was required for TTC assembly and Hcp secretion in several bacteria, including *Pseudomonas aeruginosa*, *Escherichia coli*, *Agrobacterium tumefaciens,* and *Vibrio* cholerae (19, 30, 34, 35). However, as the breadth of known T6SS subtypes increased, TssA was often found to be absent in T6SS loci, notably in all T6SS*^iii^* of the Bacteroidales and T6SS*^ii^* of Francisella, indicating it may not be a true core component. We classify TssA here within the Firing Coordinators category of TAGs due to its activity in regulating the TTC, both directly and alongside other TAG binding partners.

TssA proteins contain a conserved N-terminal ImpA_N domain (PFAM: PF06812) (Nt1), a middle domain (Nt2), and an often highly variable C-terminal domain (CTD) (36). A high-level view of TssA function is in facilitating polymerization of the TTC from the BP, and in some cases, contributing directly to the dynamics of TTC contraction (31, 32, 34, 37). The Nt1 domain dictates specific interactions with the TTC, while the CTD determines oligomerization state (33). Generally, TssA primes TTC initiation at the BP, and as TTC subunits are added to the growing sheath-tube, TssA progresses toward the opposite cell membrane with the distal end of the TTC. However, the details of a particular TssA homolog’s activity appear to be greatly influenced by interactions between its Nt1 and cognate TAG, discussed in more detail in the “Firing Coordinator” section below (32, 33).

TssA homologs have been categorized using three different approaches. The first was driven by structural and functional differences between the *E. coli* TssA (from enteroaggregative *E. coli* [EAEC]) and *P. aeruginosa* TssA1 (from the *P. aeruginosa* T6SS-H1), the earliest TssA homologs to be thoroughly investigated. EAEC TssA CTD forms a dodecameric, six-pointed star, and has a comparable size to the Hcp hexamers with which it interacts (34). *P. aeruginosa* TssA1 likewise forms ring structures, with some variability in oligomerization state (30). Reviewing recent TssA findings in 2017, Zoued et al. noted that the two TssA homologs have distinct C-terminal domains, binding partners, and relative contributions to T6SS secretion (38). As such, they separated the EAEC TssA and *P. aeruginosa* TssA1 into separate classes. Expanding on that observation, Dix et al. reinforced the two clades of TssA (1 and 2) while defining two subclades for each (A and B) (36). In their classification, EAEC TssA falls in TssA2^B^, while the *P. aeruginosa* TssA1 is in TssA1^A^. Importantly, the authors noted that members of the TssA1 clade only have two domains, Nt1 and CTD, joined by linkers, while clade TssA2 has all three domains. In addition, TssA1 has large CTD rings with many subunits, compared to TssA2’s smaller CTD rings and fewer subunits. Further work has revealed that TssA1_A_ has sixfold symmetry, TssA1_B_ are dotriacontamers (32 monomers) with 16-fold symmetry, TssA2_B_ are dodecamers with sixfold symmetry, and TssA2_A_ are decamers with fivefold symmetry (39).

The second approach for categorizing TssA was motivated by the functional roles of TssA homologs, and resulted in the classes TsaC (Class A, T6SS Sheath Assembly Chaperone), TsmA (Class B, T6SS Sheath Membrane Anchor), and TsaB (Class C, T6SS Sheath Assembly Baseplate) (40). The EAEC TssA and *P. aeruginosa* TssA2 (from the *P. aeruginosa* T6SS-H2) were assigned to Class A, TssA homologs that enhance T6SS killing and TTC stability by both facilitating rapid TTC polymerization and forming a stable cap at the distal end of the TTC. Class B included the TssA-like proteins TagA from *V. cholerae* and *E. coli,* discussed further below. Class C was characterized by TssA1 and TssA3 (from *P. aeruginosa* T6SS-H1 and -H3), which co-localize with TTC initiation sites at the BP to prime assembly, rather than polymerization.

The third approach for categorizing TssA focused on the gene lengths, “short” or “long,” and corresponding protein sizes, resulting in the nomenclature TssA_S_ and TssA_L_ (32). TssA_S_ proteins are ~40 kDa, and include the *P. aeruginosa* TssA1 and TssA3 (from T6SS-H1 and -H3), *Pseudomonas putida* TssA (from *P. putida* K1-T6SS), and *Burkholderia cenocepacia* TssA1 (from *Burkholderia* T6SS-1) homologs. TssA_L_ proteins are 55 to 60 kDa, contain the Nt2 domain, and include the EAEC, *P. aeruginosa* TssA2 (from T6SS-H2), *Burkholderia* TssA5 (from *Burkholderia* T6SS-5), *V. cholerae* TssA*,* and *Aeromonas hydrophila* TssA homologs.

When fully assembled, the TTC extends downward from the BP through the cytoplasm, often contacting the opposite end of the cell envelope before reaching full extension (27, 31, 37, 41). Upon stimulus to fire, the BP changes conformation, prompting a widening of the MC’s inner pore (17, 18). A linker between heterodimers of the sheath then rapidly contracts, propelling the TTC spike and tail through the MC pore (42). The effectors, tail, and spike proteins are quickly delivered to the target recipient, where the effectors target critical, conserved cellular structures (25, 27, 43). T6SS intoxication remains highly effective, avoiding evolved resistance in recipient cells by delivering multiple effector toxins with different molecular targets and circumnavigating the need for receptors (9, 44). Effector delivery is bacteriostatic or bactericidal depending on the specific toxin or synergistic combination of toxins (9, 45–49). Following contraction, the TTC is recycled by a hexameric AAA+ ATPase (ClpV, or “TssH”), allowing for the MC and BP to be re-used for subsequent firing with a newly polymerized TTC (7, 32, 50–53).

The T6SS has been classified into four distinct taxonomic clades based on locus composition, referred to as T6SS*^i–iv^*. T6SS*^i^* is restrained to phylum Pseudomonadota (7), T6SS*^ii^* to the Pseudomonadota genus *Francisella* (11, 54–56), T6SS*^iii^* to the phylum Bacteroidota (20, 57), and T6SS*^iv^* to the Bacteroidota genus *Amoebophilus* (58). While each clade displays heterogeneity in subcomplex composition and unique effectors, the major functional roles of each subcomplex are conserved across phyla.

**Fig 1:**
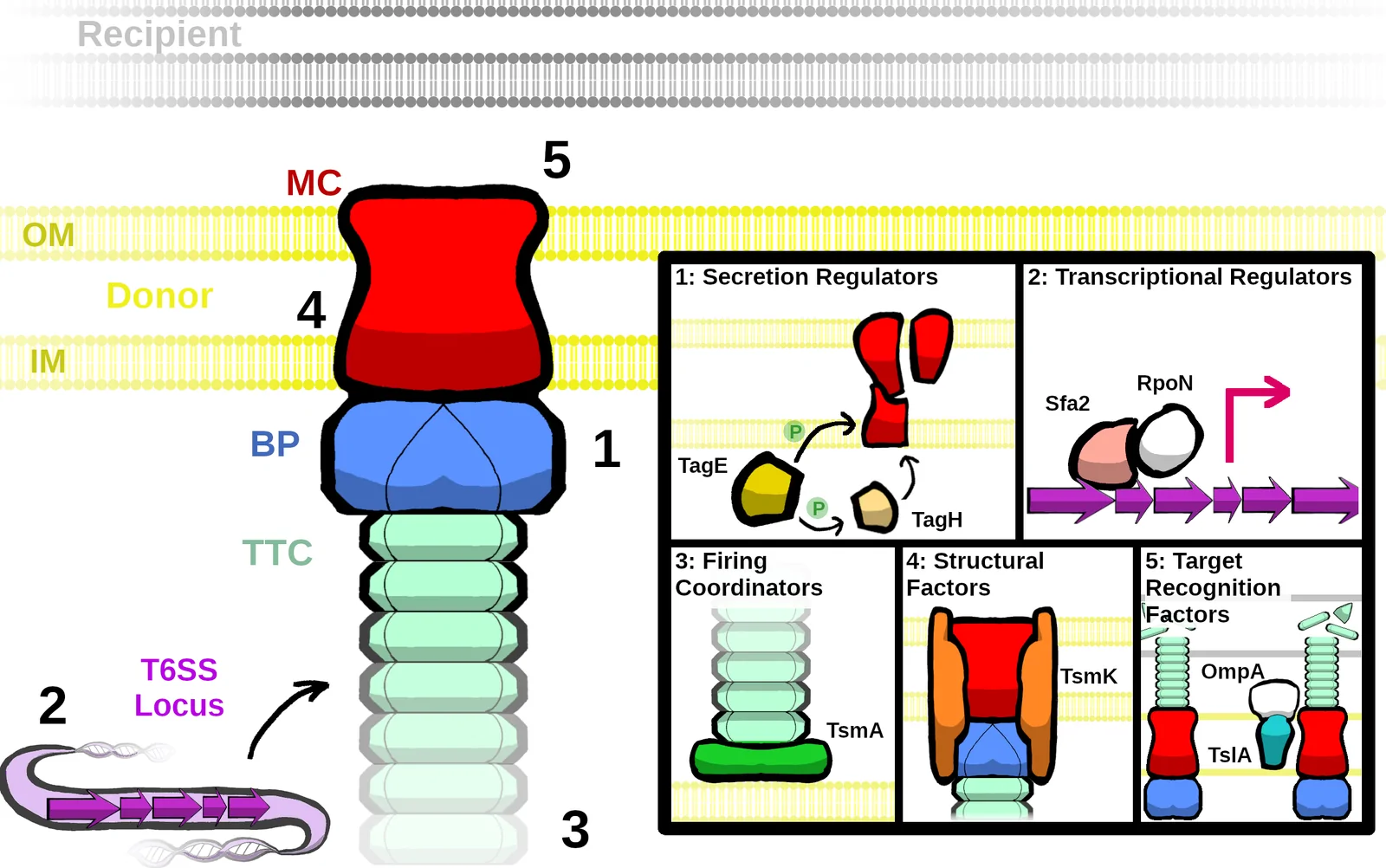
Functional classes of TAGs. TAGs expressed by a T6SS-encoding (donor) cell fall within six classes. (1) Secretion Regulators, (2) Transcriptional Regulators, (3) Firing Coordinators, (4) Structural Factors, (5) Target Recognition Factors, and Unknown TAGs (not shown). OM, outer membrane; IM, inner membrane; MC, membrane complex; BP, baseplate; TTC, tail-tube complex.

In contrast, T6SS accessory factors appear to be widely variable in terms of structure and role yet may modify functionally similar aspects of the apparatus. Depending on the accessory factor and T6SS system, it can either function to enhance the activity of the T6SS, or can be strictly required. Here we review these accessory factors, or TAGs, hoping to consolidate disparate TAGs into functionally related categories, as well as shed light on areas of T6SS regulation that require further research. We find that TAGs can be classified into six categories: Firing Coordinators, Target Recognition Factors, Secretion Regulators, Transcriptional Regulators, Structural Factors, and currently Unknown Regulators (Fig. 1; Table 1).

**TABLE 1:** TAG functional categories^*a*^

Protein	Other names	Genera containing	Confirmed binding partners	Function	Functional category	Citation
ACIAD2685	Hyp2, ACX60_11650, DSM30011_11465	*Acinetobacter*		Mechanism unknown. Predicted membrane protein with N and C termini in the cytoplasm. Required for sheath assembly, Hcp secretion, and full killing of *E. coli* recipients. Possibly a homolog of *tagZ*	Unknown Regulator	(59–61)
RtkS (regulator of T6SS kinase in Serratia)	SMDB11_2269	*Erwinia* (*2*)*, Pantoea* (*2*)*, Serratia*	TagE (periplasmic domain)	Substitute for TagQR as upstream regulator of TagE. Induces T6SS assembly through TPP pathway	Secretion Regulator (diverse)	(62)
Sfa2	VasH2	*Aeromonas*, *Pseudomonas* (*H2*), *Vibrio*	RpoN (Sigma54)	Sigma factor activator/enhancer binding protein. Enhances RpoN sigma factor binding. Coordinates control of the H2-T6SS and H2 effector islands through RpoN activation. Represses H1- and H3-T6SS. In *Vibrio*, encoded in VAC cluster. Shown to enhance expression of T6SS when Hcp levels are low and repress when Hcp is high	Transcriptional Regulator (T6SS loci)	(8, 63–66)
Sfa3	SfnR2	*Pseudomonas* (*H3*)		Mechanism unknown. Has not been shown to control H3-T6SS genes, and cannot rescue loss of Sfa2	Unknown Regulator	(65)
TagA (Bacteroidota specific)		*Bacteroides* (*GA1, GA2*)		Unknown. Predicted homology to Tfp pilus assembly protein PilF	Unknown Regulator	(67, 68)
TagA/B (Burkholderia specific)	BPSS1504	*Burkholderia* (*3, 4, 5*)		Contains an N-terminal DUF2169, while C-terminus is a pentapeptide repeat protein domain, suggested to be a “Multi-Domain DUF2169,” of Synteny type C. Likely binds PAAR-like PIPY domain for VgrG secretion. Required for Hcp-5 secretion and full virulence in mice. Also required for sheath assembly	PAAR-like	(10, 69–73)
TagB (Bacteroidota specific)		*Bacteroides* (*GA1*)		Mechanism unknown. Contributes to full Hcp secretion. Predicted pentatricopeptide domain containing protein	Unknown Regulator	(67, 68)
TagB (Burkholderia specific)		*Burkholderia* (*3, 4, 5*)		Mechanism unknown. Required for sheath assembly	Unknown Regulator	(10, 73)
TagC (Bacteroidota specific)		*Bacteroides* (*GA3*)		Unknown	Unknown Regulator	(67)
TagC (Burkholderia specific)		*Burkholderia* (*3, 4, 5*)		Mechanism unknown. Contains DUF3540. Required for sheath assembly	Unknown Regulator	(10, 73, 74)
TagD		*Burkholderia* (*3, 4, 5*)		Mechanism unknown. PAAR-like but no effector domain, defined as PAAR_E clade with DUF4150 domain. Required for sheath assembly and contributes to dissemination during cell-cell fusion. Deletion reduces levels of VgrG5	PAAR-like	(10, 73–76)
TagE	PpkA, PknA, PA0074	*Agrobacterium*, *Azoarcus* (*2*), *Pantoea* (*2*), *Pseudomonas* (*H1*), *Burkholderia* (*6?*), *Serratia*, *Xanthomonas*		Serine-threonine protein kinase. Activated by TagR to homodimerize and autophosphorylate. Phosphorylates TagH downstream to activate the T6SS. Required for full Hcp secretion. Shown in *Agrobacterium* to also phosphorylate TssL to aid Hcp secretion	Secretion Regulator (diverse)	(77–82)
TagF	SciT, ImpM, PA0076	*Acinetobacter* (*1A*, *1B*, *2*), *Achromobacter*, *Agrobacterium*, *Azoarcus* (*2*), *Burkholderia* (*1*, *5*, *6*), *Neisseria* (*B*), *Pantoea* (*1*, *2*), *Pseudomonas* (*H1*, *K1*), *Rhizobium*, *Salmonella*, *Serratia*, *Xanthomonas*	TagH	Inhibits membrane complex assembly by sequestering TagH. Not required for sheath localization or assembly	Secretion Regulator (diverse)	(59, 62, 79, 81, 83–87)
TagG	PppA, PA0075	*Azoarcus* (*2*), *Pseudomonas* (*H1*), *Pantoea* (*2*), *Serratia*, *Xanthomonas*		Threonine protein phosphatase. Dephosphorylates TagE to inhibit T6SS assembly	Secretion Regulator (diverse)	(62, 77, 78, 82)
TagH	ImpI, Fha1, VCA0112, PA0081, SMA2267, PSPTO_5422	*Aeromonas*, *Agrobacterium*, *Azoarcus* (*2*), *Burkholderia* (*6*), *Pantoea* (*2*), *Pseudomonas* (*H1*, *H2*), *Serratia*, *Vibrio*	TagF, ClpV (Pseudomonas), p-TssL (Agrobacterium)	Forkhead-associated domain protein. Activated by TagE to promote T6SS assembly. Repressed by TagF	Secretion Regulator (diverse)	(10, 48, 62, 66, 77–81, 88, 89)
TagI		*Burkholderia* (*6*)		Putative MotB/OmpA-like peptidoglycan binding lipoprotein	Unknown Regulator	(10)
TagJ	HsiE1, ImpE, SciE	*Agrobacterium*, *Azoarcis* (*2*), *Burkholderia* (*2*, *3*), *Neisseria* (*B*), *Pseudomonas* (*H1*), *Salmonella*, *Serratia*, *Xanthomonas*	TssA, TssB, ClpV	Similar role to TsmB, though not homologous. Allows for stable sheaths. Originally proposed to be involved in ClpV recycling activity	Firing Coordinator (TTC)	(32, 52, 84, 87, 90)
TagK	*xac4148*	*Burkholderia* (*2*, *3*), *Caulobacterales*, *Halomonas*, *Oceanospirillales*, *Salmonella*, *Xanthomonas*		Unknown in *Burkholderia*, shown in *Xanthomonas* to be an AraC-family transcriptional regulator of T6SS genes	Transcriptional Regulator (T6SS loci)	(10, 91)
TagL	SciZ	*Escherichia*, *Burkholderia* (*2*, *7*), *Klebsiella* (*locus I*, *II*, *and III*), *Neisseria* (*A*), *Pseudomonas* (*Putida*), *Ralstonia*, *Yersinia*	TssL (transmembrane domains only)	Anchors T6SS to cell wall when TssL lacks a peptidoglycan binding domain. Localizes to MC after TssJLM assemble and MltE has remodeled the cell wall. Necessary for sheath assembly after BP docking	Structural Factor (MC and BP)	(37, 83, 84, 92–95)
TagM1	BTH_I2965	*Burkholderia* (*1*), *Xanthomonas*, *Achromobacter*		Putative outer membrane-anchored lipoprotein. Likely a homolog of TslA	Target Recognition Factor (donor-recipient)	(10, 95, 96)
TagN	ACIAD2682, ACX60_11635	*Acinetobacter* (*1A*, *1B*), *Achromobacter*, *Burkholderia* (*1*, *5*), *Xanthomonas*		Putative peptidoglycan-binding protein. Unclear impact on Hcp secretion, competition, and TTC dynamics	Unknown Regulator	(59, 61, 92, 95–97)
TagO	NOG245466, HMPREF1171_00941, VasI	*Aeromonas*, *Pseudomonas* (*H4*)		Unknown	Unknown Regulator	(98, 99)
TagP	PP3090	*Pseudomonas* (*putida*)		Contains a peptidoglycan-binding domain, putative homolog of TssM. Predicted inner membrane localization	Unknown Regulator	(83, 92, 100)
TagP (PAAR-C)	ACX60_RS09070	*Acinetobacter*		PAAR gene referred to as TagP. Located in auxiliary PAAR cluster, not within main T6SS locus	Core Component	(100)
TagQ		*Pseudomonas* (*H1*)		OM Lipoprotein that interacts with TagR to phosphorylate TagTS at initiation of TPP pathway. Results in T6SS assembly at sites of membrane damage and/or T6SS attack	Target Recognition Factor (donor-recipient)	(43, 89, 101, 102)
TagR	COG1262	*Pseudomonas* (*H1*), *Rhodobacter*		Periplasmic protein that is activated by TagQ to allow phosphorylation of TagTS during TPP pathway. Results in T6SS assembly at sites of membrane damage and/or T6SS attack	Target Recognition Factor (donor-recipient)	(43, 78, 89, 101, 102)
TagS	COG4591	*Pseudomonas* (*H1*), *Rhodobacter*		Part of ABC-transporter-like complex TagTS. Phosphorylated by TagQR to activate TagE. Results in T6SS assembly at sites of membrane damage and/or T6SS attack	Target Recognition Factor (donor-recipient)	(43, 89, 101, 102)
TagT	COG1136	*Pseudomonas* (*H1*), *Rhodobacter*		Part of ABC-transporter-like complex TagTS. Phosphorylated by TagQR to activate TagE. Results in T6SS assembly at sites of membrane damage and/or T6SS attack	Target Recognition Factor (donor-recipient)	(43, 89, 101, 102)
TagU	KX941486	*Pseudomonas*		Predicted lipoprotein of unknown function	Unknown Regulator	(103, 104)
TagV	SMDB11_2251	*Serratia*, *Pantoea*, *Erwinia*, *Cronobacter*	Does not bind TssJ, TssL, or TssM, and no binding partners identified through co-purification pulldown and mass spectrometry	Outer membrane lipoprotein required for full competition and Hcp secretion. Allows for some T6SS activity even in the absence of TssJ	Structural Factor (MC and BP)	(69)
TagW		*Vibrio*		Unknown. Predicted to contain a peptidoglycan-binding domain	Unknown Regulator	(92)
TagX	AsaE, ACIAD2699, ACX60_11600, A1S_1311_12, BCAL0352, PP3100.1	*Achromobacter*, *Acinetobacter* (*1A*, *1B*), *Burkholderia* (*1*), *Ralstonia*, *Pseudomonas* (*putida*), *Xanthomonas*	TssL, TssM, TsmK, TslA	Endopeptidase that allows for T6SS localization, activity, Hcp secretion, and prey killing. Not strictly required for sheath initiation	Secretion Regulator (diverse)	(59, 61, 83, 95–97, 105)
TagY	BCAL0353	*Burkholderia* (*1*), *Achromobacter*, *Xanthomonas*		Unknown. Predicted to have a short cytoplasmic domain, TMD, and periplasmic domain with possible metal ion binding. Not required for Hcp secretion	Unknown Regulator	(95)
TagZ	BCAL1353	*Achromobacter*, *Acinetobacter*, *Burkholderia* (*1*), *Ralstonia*, *Rhodocyclus*, *Xanthomonas*		Unknown. Predicted to have a short cytoplasmic domain, TMD, and periplasmic domain with possible metal ion binding. Often found encoded next to a PAAR gene	Unknown Regulator	(95)
TasL	VFES401_15750	*Vibrio* (*T6SS2*)		Predicted outer membrane lipoprotein with a plasma fibronectin type III-like domain outside the cell. Required for killing in mucus-imitating hydrogel, in *E. scolopes* squid light organs, but not on agar surfaces. Enhances cell-cell contact to facilitate optimal T6SS killing	Target Recognition Factor (donor-recipient)	(106)
TsaB	TssA1 and TssA3 (short), SciA, ImpA, VasJ, HsiA	*Escherichia*, *Pseudomonas* (*H1*, *H3*), *Vibrio*, *Salmonella*, *Yersinia*, *Burkholderia* (*1*), *Serratia*, *Acinetobacter*, *Agrobacterium*	TssL, TssM, TssK, TssE, TssF, TssC, VgrG, TssB, Hcp, ClpV (Pseudomonas TssA1)	Activator of TTC assembly at BP. Forms dodecameric ring	Firing Coordinator (TTC)	(19, 30–40)
TsaC	TssA2 (long), SciA, ImpA, VasJ, HsiA	*Escherichia*, *Pseudomonas* (*H2*), *Vibrio*, *Salmonella*, *Yersinia*, *Burkholderia* (*5*), *Serratia*, *Aeromonas*	TssL, TssJ, TssM, TssK, TssE, TssF, TssG, TssK, VgrG, TssB, TssC, Hcp (TssA2 in EAEC), TagA/TsmA	Distal end chaperone. TssA2 (long) interacts with MC, triggering BP recruitment, then coordinates TTC assembly while traveling distally. TssA2 binds TagA at opposite end of the cell, controls firing dynamics by maintaining elongated sheath	Firing Coordinator (TTC)	(19, 30–40)
TslA	AsaA, ACIAD2693, A1S_1292, ACX60_11685, DMS30011_11500, Hyp1	*Acinetobacter*	TssM, TagX	Periplasmic protein that initiates membrane complex assembly following cell-cell contact. Mediated by OmpA in the target recipient	Target Recognition Factor (donor-recipient)	(59–61, 96, 97, 107, 108)
TsmA	TagA	*Aeromonas*, *Escherichia*, *Photorhabdus*, *Vibrio*, *Xenorhabdus*	TssA/TsmC, TssL, TssM, TssE, TssF, TssK, VgrG, TssB, TssC, Hcp (Vibrio)	Primes sheath initiation alongside TssA/TsmC. Localizes to the cell membrane opposite the T6SS and prevents excessive sheath assembly by interacting with TssA/TsmC. Controls sheath length and allows for extended periods of polymerized sheaths before firing	Firing Coordinator (TTC)	(31, 37, 109, 110)
TsmB	TagB1, TagX1	*Pseudomonas*	TssA, TssK, TssE, TssF, VgrG, Hcp	Localizes to baseplate during initiation, allows for fully extended, stable sheaths. May form a collar around the baseplate to prevent the conformational change that potentiates firing	Firing Coordinator (TTC)	(32, 69)
TsmK	AsaB, A1S_1301	*Acinetobacter*	TssB, TssC, TssF, TssK, TssL, TssM, TagX, OmpA, CarO	Stabilizes the TssL-TssM MC and interacts with TagX and TslA for localization of the T6SS. Aids anchoring to the BP during assembly	Structural Factor (MC and BP)	(97)
VirA	BMAA0746, BPSS 1495	*Burkholderia* (*5*)		Sensor histidine kinase of two-component system. Transcription is activated by BsaN/BicA. Senses host low molecular weight thiols during infection. Activates VirG	Transcriptional Regulator (T6SS loci)	(111–117)
VirG		*Burkholderia* (*5*)		Response regulator of two-component system. Activated by VirA to drive transcription of T6SS	Transcriptional Regulator (T6SS loci)	(111–117)

^*a*^ TAGs are organized alphabetically and color-coded by functional category.

## RESULTS

As bacteria compete for limited resources and niches, the T6SS provides a competitive advantage against neighboring cells. However, bacteria must balance T6SS activity with its cost, which often manifests during *in vivo* conditions and may be due to expression of the numerous core proteins, increased interbacterial antagonism, or unproductive firing against kin cells (118–121). TAGs optimize T6SS functionality by minimizing costs and maximizing productive T6SS firing. Though their presence, structure, and location in T6SS loci vary across the diverse taxa that employ them, TAGs tend to interact at similar critical steps. Therefore, we have sorted TAGs into six categories, which include both the role and the point at which the TAG interacts. We hope to provide organization to a diverse body of literature, as well as a guiding framework for investigating the roles of novel TAGs.

### Firing Coordinators (TTC interactors)

Some of the most well-studied TAGs fall within the category of Firing Coordinators, which optimize TTC assembly, contraction, and secretion. The Firing Coordinators discovered thus far interact primarily with the TTC proteins, such as the sheath components TssB and TssC, but can also have interactions with the MC and BP. These TAGs include TssA (Box 1), TsmA (TagA), TsmB (TagB/TagX1), and TagJ (HsiE).

TsmA was first discovered while studying TssA homologs (38). It was initially considered a member of the TssA family, as it contains an N-terminal ImpA (ImpA_N) domain, and was named Type VI Associated Gene with ImpA domain (TagA). As mentioned above (Box 1), further classification attempts regarding TssA homology were based on three functional classes; Class B included TagA proteins, thereafter referred to as T6SS Sheath Membrane Anchor (TsmA) (40).

A proximity biotinylation mass spectrometry experiment in EAEC found that TsmA was a close partner with TssA (109). Deletion of the MC or BP components, required for TssA to initiate TTC polymerization, abolished the TssA-TsmA interaction, providing evidence that TsmA binds TssA at the end stage of its TTC processing. True to its role as an accessory factor, deletion of *tagA* still allowed competition between donor EAEC and recipient *E. coli* K-12, but at a reduced efficiency (12% that of wild type). TsmA was found to localize in the cytoplasm, but associate with the membrane independent of the T6SS. In *V. cholerae*, TsmA, alongside TssA were shown to contribute to TTC priming, and TsmA bound a myriad of MC, BP, and TTC subunits (31). Deletion of *tsmA* yielded a striking phenotype, whereby the TTC sheath would polymerize to the opposite cell membrane from the MC and BP, and in ~50% of cases, continue extending parallel with the membrane (109, 110). This observation, paired with data showing the localization of TsmA to the distal end of fully polymerized sheaths, implicated TsmA as a terminator signifying completed TTC assembly (37). Interestingly, in more than 60% of cells with extended sheaths, *tsmA*-deficient cells immediately fired the T6SS and contracted their TTC (109, 110, 122). The evidence supports TsmA as an accessory factor that both coordinates proper TTC assembly and stabilizes polymerized sheaths until the cell is prepared to fire.

The TAG TsmB was first identified in the *P. putida* K1-T6SS, where it was named TagX1 (PP3100.1 or PP_5562) (83). It was later renamed TagB to better illustrate its related functional role to TagA (TsmA) in Firing Coordination. To reflect its role as a regulator of the TTC with TssA_S_ (TsaB), TagX1/TagB was renamed TsmB, a nomenclature we adopt (69). As TssA_L_ was found to operate in conjunction with TsmA, likewise, TssA_S_ has an interaction partner in TsmB. TsmB bound to TssA, and *tssA* deletion cells lost TsmB-sfGFP puncta (32). TsmB was found to interact with many of the BP components via bacterial two-hybrid assays, and TsmB-sfGFP localized at the proximal end of polymerizing sheaths, supporting an interaction with TssA at the BP. Upon sheath contraction, TsmB-sfGFP signal dissipated. Consistent with an accessory role, deletion of TsmB did not significantly reduce the number of cells with polymerized sheaths; however, sheath lengths were ~50% of the length compared to cells with TsmB, and cells lacking *tsmB* had reduced interbacterial competition against recipient strains. Across different T6SS subtypes, the cognate TAG for TssA_S_ or TssA_L_ directly impacts firing coordination by controlling “residence time,” the time after full TTC polymerization and before contraction. While cells expressing TssA_L_, coordinated by TsmA, can have T6SS residence times of longer than 10 min, cells expressing TssA_S_, coordinated by TsmB, fire shortly after reaching full TTC lengths (32, 40, 109). Altogether, firing coordination by TsmB assists interbacterial competition by facilitating optimal TTC architecture.

Another related Firing Coordinator is the protein TagJ, which was first described in *P. aeruginosa* T6SS-H1 and named hsiE1 (90). TagJ interacts with the TTC sheath component TssB, but deletion of *tagJ* does not inhibit T6SS secretion. Bacterial two-hybrid analysis revealed ClpV as a strong interactor with TagJ, prompting a model whereby TagJ recruits ClpV to the sheath to recycle the TTC (52). TagJ was later found to interact in dot blot assays with TssA_S_ from the T6SS-H1, similar to TsmB (32). In addition, TagJ-sfGFP molecules imitate TsmB-sfGFP, transiently switching from diffuse to stable foci. Though TagJ lacks sequence similarity to TsmB, it is currently considered a functionally related binding partner for TssA_S_ homologs and may also coordinate T6SS firing.

### Target Recognition Factors (donor-recipient interface)

Target Recognition Factors are T6SS accessory proteins that facilitate the interaction between donors and their target recipients. As they function at the cell-cell interface, they can interact with numerous proteins, including both components of the donor T6SS and the recipient membrane proteins. Here, we have classified the TAGs TslA (AsaA, Hyp1), TagM1, and TagQRST as Target Recognition Factors.

Early work describing the T6SS in *Acinetobacter baumannii* referred to the gene immediately upstream of *tssB* and *tssC* as Acinetobacter type six secretion system-Associated (*asaA*)*,* as it had no known T6SS role or homologs outside of *Acinetobacter* at the time (107). To discover the roles of the genes in the *Acinetobacter* T6SS locus, researchers took advantage of the fact that many *Acinetobacter* strains carry plasmids that both transcriptionally repress the chromosomal T6SS genes and encode antibiotic resistance genes (123). They compared RNA-seq data of *A. baumannii* with and without the repressor plasmid (59). Among other upregulated T6SS genes, *asaA* was 46-fold more abundantly expressed when the T6SS was derepressed. AsaA was predicted to localize to the periplasm as an outer membrane-bound lipoprotein (60). Deletion of *asaA* had a variable effect on Hcp secretion, with some labs finding reduced secretion and others no obvious effect (59, 61, 97, 108). Further indicating its role as an accessory rather than core component, *asaA*’s deletion only moderately reduced the growth of recipient *E. coli* cells competing with donor *A. baumannii* (61, 97, 108). Cellular fractionation of AsaA-6x-HIS revealed a periplasmic localization, where it interacted with the periplasmic portion of the MC component TssM (108). Later work described a full protein-protein interaction network, in which AsaA-FLAG binds TagX-VSVG, which then binds the MC components (97). AsaA appears to contribute to MC stability, as removal of *asaA* also reduces the average number of MC foci from ~1/cell to ~0.4/cell (97).

AsaA was renamed Type VI Secretion system dynamic Localization protein A (TslA), following studies investigating the localization of the TTC in *Acinetobacter* (96). Researchers noticed that ~33% of TTCs formed at cell-cell contact sites. Surprisingly, stable TTCs formed at these sites even in non-functional T6SS mutant strains. Deletion of *tslA* in donor *Acinetobacter baylyi* removed the cell-cell contact TTCs. The authors therefore proposed TslA-mediated target recognition at cell-cell connection sites. Interestingly, deletion of *ompA* also reduced T6SS assembly at cell-cell sites. In combination, the data support TslA as a periplasmic protein that, in conjunction with the outer membrane protein OmpA, recognizes target cells. TslA then supports MC stability at contact sites with targets, leading to localized TTC assembly and successful delivery of toxin effectors.

Interestingly, TslA homologs can bear markedly diverse sequences while still retaining similar function: *tslA* in *A. baylyi* and *A. baumannii* share only 48% sequence identity (96). TagM1 is likely a homolog of TslA found in *Burkholderia*, among other genera. TagM1 was first found in *B. pseudomallei*, then *B. cenocepacia,* then *B. thailandensis* (10, 95, 96). Similar to TslA, TagM1 was predicted to be localized to the periplasm as an outer membrane-anchored lipoprotein. Deletion of *tagM1* in *B. thailandensis* nearly abolished cell-cell contact TTC assembly and had a very strong inhibitory effect on the killing of target *E. coli* (96). Though it appears to be involved in recognizing target cells, more work needs to be done to determine the exact role of TagM1 in facilitating T6SS activity.

Mentioned in more detail below, the Threonine Phosphorylation Pathway (TPP, or “TagQRST-PpkA-Fha1-PppA”) can be triggered by the Target Recognition Factors *tagQRST*. TagQ, an outer membrane lipoprotein, functions as the sensor module, which becomes activated at sites of membrane damage and/or T6SS attack (88, 101, 102). TagQ interacts with TagR, a periplasmic protein, which phosphorylates TagTS. TagTS forms an ABC-transporter-like complex, which in turn activates the cytoplasmic effectors of the TPP. In full, TagQRST promotes T6SS assembly at cell-cell interfaces to efficiently target recipient cells for attack.

### Structural Factors (MC and BP interactors)

Accessory genes that transcribe components which reinforce the architecture of the T6SS nanomachine are Structural Factors. These TAGs often compensate for variations in the MC or BP. The Structural Factors TagL (SciZ) and TsmK (AsaB) interact with the MC and/or BP. The third Structural Factor we discuss here, TagV, currently remains a mystery regarding its point of interaction. However, the data support TagV as a Structural Factor that compensates for deletion of TssJ through an unknown mechanism.

The TAG TagL was first described in the EAEC T6SS sci-1 cluster, where *tagL* was known as *sciZ*. TagL serves as an example of a TAG that is required for T6SS activity rather than just supportive, as deletion of *tagL* blocks Hcp secretion in EAEC (92). Co-precipitation experiments with His-TagL and HA-labeled T6SS components found that TagL had a direct interaction with the MC protein TssL (SciP) and indirect interactions with the other two MC proteins, TssJ (SciN) and TssM (SciS), mediated by TssL. TagL contains three N-terminal transmembrane domains and localizes to the inner membrane with its C-terminus extending into the periplasm (93). The N-terminus alone interacts with TssL, while the C-terminus was predicted to contain a peptidoglycan-binding domain of the OmpA/Pal/MotB family (PF00691) (10, 92, 93, 124). TagL’s ability to bind peptidoglycan was validated by DSP cross-linking experiments. Interestingly, the peptidoglycan-binding domain was also required for Hcp secretion and was sufficient to reconstitute *tagL’s* deletion when fused to TssL (92, 124). Fluorescent sfGFP-TagL frequently co-localized with mCherry-TssL and was positioned at the initiating sites of TssB-mCherry (124). Deletion of the BP components had no effect on TagL’s localization. Therefore, microscopy data further validate the role of TagL as an interactor at the MC before the MC-BP docking. Interestingly, the membrane-bound lytic transglycosylase E (MltE) protein was also required for TagL to localize to the MC. Supporting TagL’s role as an accessory to the T6SS, deletion of *tagL* did not impair the formation of the MC and BP docking, though TTC sheath polymerization was impaired. Further work observed that the majority of T6SS loci carry TssL variants (“evolved,” or “specialized,” “TssL”) containing their own peptidoglycan-binding domains. In some cases, the TssL peptidoglycan-binding domain was absent, but these T6SS loci instead contained *tagL* (84, 124). As an accessory Structural Factor, therefore, TagL supplements the peptidoglycan-binding of TssL, allowing the MC to be properly rooted in the periplasm, which has downstream effects on the ability of the TTC to polymerize. In T6SS lacking evolved TssL, TagL likely provides the peptidoglycan binding ability. Ultimately, TagL is a TAG that is required for T6SS activity in EAEC.

TsmK is a TAG present in *Acinetobacter* T6SS loci, where it was first named *asaB*. It was renamed Type VI secretion membrane complex assembly pseudo-Ketoacyl synthase (TsmK) due to its predicted structural homology to β-ketoacyl synthase (97). Deletion of *tsmK* yielded a strong inhibitory effect on Hcp secretion and T6SS-mediated killing of *E. coli*. When visualized *via* fluorescence microscopy, the inhibitory phenotype stemmed from a reduction in total MC assembly and dynamic movement in the membrane. TsmK exhibited numerous protein-protein interactions, including with MC components (TssM, TssL), BP components (TssF, TssK), TTC components (TssB, TssC, ClpV), other TAGs (TagX), and outer membrane proteins (OmpA and CarO homologs). A taxonomic analysis found that 70% of *tsmK* homologs were present only in the *Acinetobacter* genus, with high sequence variability for the remaining 30% of homologs outside of *Acinetobacter*. Intriguingly, *tsmK* was never found in a T6SS locus containing *tssJ*. The *Acinetobacter* MC appears to have a unique composition, whereby TsmK compensates for the missing TssJ as a Structural Factor TAG. Though unrelated to the TAGs discussed here, the *Acinetobacter* TssM also contains a C-terminal GS-linker, which likely further compensates for the lack of TssJ for the *Acinetobacter* MC.

An intriguing accessory factor is TagV. Though first described in the *Serratia marcescens* T6SS locus, *tagV* homologs are found in several other genera, including *Cronobacter*, *Erwinia*, and *Pantoea* (69). TagV localized to the outer membrane as a lipoprotein when assayed with sucrose density gradient fractionation. It is also predicted to contain a bacterial SH3 domain (SH3b), implying a role in binding peptidoglycan. Cells lacking *tagV* had significant reductions in Hcp secretion and T6SS-mediated competition against *P. fluorescens*. Interestingly, *tagV* deletion in competitions could be partially complemented by overexpression of TssJ, implying a related functional role. Furthermore, *tagV* lacking cells had lower levels of expressed TssJ, even when TagV was ectopically complemented. A piece of data supporting a link between TssJ and TagV came when deleting *tssJ*. In *S. marcescens*, unlike other bacteria, *tssJ* deletion does not completely abolish T6SS competition or Hcp secretion; deletion of the BP component, *tssE,* does. Surprisingly, a dual mutant of *tssJ-tagV* inhibited competition to the null level of a *tssE* deletion strain. Despite this complementary role, recombinant TagV was not found to interact with the other MC components, nor with TssJ, via size exclusion chromatography. A pull-down approach with total cell lysates analyzed by label-free quantitative mass spectrometry likewise yielded no binding partners for TagV-His. TagV therefore has an accessory role in T6SS function, orthologous to TssJ, but its exact interaction with the T6SS is still unknown. Theoretically, it may interact with the T6SS transiently, only in the context of an intact cell, or following a signal that remains to be determined. Perhaps the most fascinating discovery about TagV is that it facilitates a degree of T6SS activity even in the absence of TssJ, indicating it may be a Structural Factor.

### Transcriptional Regulators (inhibit or activate T6SS gene transcription)

In addition to facilitating the structural and dynamic aspects of the T6SS, some TAGs regulate the T6SS through effects on the transcription of their genes. Referred to here as Transcriptional Regulators, this category of TAGs includes Sfa2 (VasH2), TagK (Xac4148), and VirAG.

A well-studied Transcriptional Regulator is Sfa2, present in the *Pseudomonas* T6SS-H2. Sfa2 is a sigma factor that enhances RpoN (Sigma54, σ54) binding, an alternative sigma factor which frequently requires an enhancer binding protein (EBP) or a σ54 activator (SFA) protein (65, 125). Following triggering of T6SS transcription, *S*fa2 and RpoN coordinate expression of 34 of the H2-T6SS genes, as well as orphan (or auxiliary) H2-T6SS gene islands containing *hcp2* and *vgrG* homologues. Delegated to the type 1b EBP class, Sfa2 contains two AAA ATPase P-loop motifs, a GAF domain for regulation, a Sigma 54 Interact 4 domain for binding RpoN, and a helix-turn-helix domain for binding DNA (65). To counteract RpoN/Sfa2 activity, the protein RsmA post-transcriptionally represses all three T6SS in *Pseudomonas* (63). Indicating its importance in overall T6SS activity, *sfa2* deletion inhibited Hcp and VgrG secretion in *P. aeruginosa*, *P. aeruginosa* killing of *E. coli*, and secretion of Hcp in the phytopathogen *P. syringae* (65, 66).

Similar to *Pseudomonas, V. cholerae* T6SS contains an Sfa2 homolog, VasH, which also functions as an EBP for RpoN (8, 126). VasH was found to be required for the secretion of Hcp and T6SS effectors and virulence against amoebae and immortalized murine macrophages (8, 126). Though encoded in the main T6SS VAS cluster, VasH controls expression of two of the auxiliary *V. cholerae* T6SS clusters, which contain Hcp, VgrG, and distinct effectors (8, 15). Recent work discovered that VasH’s N-terminal regulator domain could sense cytoplasmic levels of Hcp (64). At high levels of Hcp, VasH inhibited expression of the auxiliary Hcp clusters, while low levels of Hcp relieved this repression. Presumably, this phenotype is driven by differential interactions with RpoN, dependent on the Hcp binding state of VasH. Therefore, nuances in the activity of Sfa2 homologs in diverse bacteria likely exist, though Sfa2 homologs ultimately function as Transcriptional Regulators for their cognate T6SS and/or auxiliary T6SS clusters.

TagK is a Transcriptional Regulator TAG first discovered in *Burkholderia*, but only mechanistically studied in *Xanthomonas* (10). The phytopathogen *X. citri* utilizes a T6SS to combat the bacteriovorous amoeba *Dictyostelium discoideum*. Upon contact with *D. discoideum, X. citri’s* Ser/Thr kinase PknS activates the alternative sigma factor EcfK via phosphorylation (127, 128). A screening of the active EcfK regulon identified TagK, an araC-family transcription factor (91). TagK exclusively binds to and activates transcription of a set of 25 genes: the *X. citri* T6SS locus and two uncharacterized genes predicted to encode a T5SS. TagK serves as a well-described T6SS Transcriptional Regulator contained within the *Xanthomonas* T6SS locus.

Located within the T6SS-5 locus, the two-component regulator VirAG acts as a Transcriptional Regulator during *Burkholderia* virulence. VirAG was first identified as part of a two-component system in *B. mallei* that was required for virulence in a hamster model of infection (111). Further experiments have shown they are also required for virulence in RAW 264.7 mouse macrophages and mouse infections (112). Transcription of VirA, the sensor, and VirG, the response regulator, is driven by BsaN/BicA, genes located in the T3SS-3 locus (115). Once transcribed, VirA is predicted to localize to the inner membrane, where its periplasmic domain can recognize host low molecular weight thiols, including glutathione (116). Glutathione was found to strongly activate VirA, causing it to become reduced, at which point it can likely activate VirG. The VirAG regulon contains ~60 genes, including the T6SS-5 genes and the virulence factor *bimA* (113). Overexpression of *virAG* in *trans* was found to be sufficient for induction of T6SS genes and Hcp secretion (114). Canonically, *Burkholderia* employ T6SS-5 for eukaryotic virulence and T6SS-1 for interbacterial competition. Surprisingly, overexpression of *virAG* also enriched substrates known to be secreted from T6SS-1 (117). VirAG is part of a larger regulatory network in *Burkholderia* that has been reviewed elsewhere (129). For our purposes, VirAG serves as a well-characterized example of Transcriptional Regulators within T6SS loci that upregulate T6SS expression only when necessary, theoretically mitigating the cost of the T6SS.

### Secretion Regulators (diverse)

Secretion Regulators modify the core T6SS components to activate and/or enhance secretion. Some TAGs regulate the T6SS without directly integrating with the T6SS architecture (as the Structural Factors) or facilitating firing via interactions with the TTC (as the Firing Coordinators). We therefore refer to these TAGs as Secretion Regulators. As three examples of Secretion Regulators, we discuss the TPP pathway agent TagE (PpkA), the instigator of T6SS assembly TagH (Fha1), and cell wall remodeler TagX (AsaE).

Shortly after the discovery of the T6SS in *P. aeruginosa* and *V. cholerae*, the regulatory pathway for T6SS activation became an area of great interest. Early characterization of the T6SS-H1 in *Pseudomonas* was aided by the fact that deletion of *retS*, a histidine kinase regulator, greatly increased Hcp secretion (7). RetS was found to be acting on the Ser-Thr kinase TagE (PpkA), present within the T6SS-H1 (7, 130). Deletion of *tagE* was sufficient to block Hcp secretion. Further work described TagE as an activator of the T6SS and the phosphatase TagG (PppA) as its reciprocal repressor (77).

Though variations in the TPP exist, the prototypical signal cascade begins with an activator triggering TagE to dimerize and autophosphorylate (78). TagE then activates a downstream signal cascade member in the cytoplasm. The endpoint of the TPP is the assembly of T6SS at specific, advantageous sites, such as cell-cell interfaces. In *Pseudomonas,* TPP is triggered at sites of membrane damage by the Target Recognition Factors TagQ, TagR, TagS, and TagT, mentioned above (43, 78, 89). In *Serratia*, TPP is instead activated by the periplasmic kinase RtkS (62). Recent data on *Klebsiella pneumoniae* found that its T6SS is active only under particularly stressful culturing conditions or in competition with other T6SS-encoding bacteria, possibly indicating a similar antagonism-activated pathway may be present in *Klebsiella* (94, 131).

Initially, activated TagE was considered to have only one phosphorylation target, the TPP effector TagH. TagH was first named Fha1 (Forkhead-associated 1) due to its forkhead-associated (FHA) domain, before being designated as a TAG (10, 77). FHA domains recognize phospho-Thr peptides on binding partners, perpetuating signal cascades in processes, including type III secretion, sporulation, and metabolism (132). True to its predicted role as an FHA signaling protein, TagH was found to bind phospho-Thr on TagE and to be a substrate for TagE-mediated phosphorylation (77, 78). Though its exact mechanism is still unclear, activated TagH is able to stimulate T6SS assembly at advantageous sites. In the *P. aeruginosa* T6SS-H1, *A. tumefaciens* T6SS, and *S. marcescens* T6SS, phosphorylated TagH is required for Hcp secretion (77, 79–81). With such a critical role, TagH is under tight control within the cell. TagG represses T6SS assembly by dephosphorylating TagH (43, 77). Unphosphorylated TagH is inhibited by sequestration *via* TagF (62). Therefore, careful coordination of TPP activators and repressors allows for dynamic assembly and rearrangement of the T6SS to combat competitors while minimizing the assembly of undesirable T6SS.

Beyond its role in activating TagH, TagE has been shown in some bacteria to phosphorylate the MC directly. In the *A. tumefaciens* T6SS system, which includes TagE, TagG, and TagH accessories, TagE phosphorylates TssL rather than TagH (79). Phosphorylation of TssL allows for a further interaction with TagH (Fha2) at the phospho-Thr peptide. This p-TssL-TagH interaction is required for full Hcp secretion.

As with many aspects of T6SS diversity, there are variations in the TPP, including TagE and TagH. The repertoire of TPP components varies, with the minimal genes considered to be *tagE*, *tagG*, and *tagH*. The TPP activators TagQRST have only been studied in *Pseudomonas*, while *tagEGH* are present in more diverse taxa (62). Some species, including *V. cholerae, E. coli* O157, and *Yersinia pestis*, still encode *tagH* in their T6SS loci while lacking *tagE* and *tagG* (133). As it lacks its activator, TagE, it appears that TagH has a different mechanism in T6SS regulation in these bacteria. In *V. cholerae*, *tagH* was found to be required for Hcp secretion regardless of lacking *tagE* and *tagG* (35, 133). Interestingly, TagH was also found to be a negative regulator of hemolytic activity on sheep blood agar (133). TagH inhibited Hemolysin A (*hlyA*) transcription, the major driver of hemolysis by *V. cholerae*. Infection of rabbit ileal loops revealed greater enterotoxicity when *tagH* was absent in the infecting strain, as did an intraperitoneal mouse infection to mimic sepsis. These phenotypes appear to be independent of T6SS activity, though *tagH* is still encoded within the T6SS locus. It may be that bacteria can co-opt T6SS TAGs for disparate activities, even if the T6SS-specific functionality is lost. Presumably, this co-opting of TAGs must be driven by the particular microbial context proving evolutionarily favorable, such as *V. cholerae* employing TagH to temper virulence during host infection.

TagX is another Structural Factor that was first well-described in *Acinetobacter* (59). While known as locus tag BCAL0352 in *Burkholderia*, it was found to be dispensable for virulence in macrophages but was required for Hcp secretion (105). In *Acinetobacter*, TagX was predicted to be localized to the periplasm, have two transmembrane domains near the N-terminus, and contain a C-terminal peptidase domain corresponding to the VanY superfamily of peptidoglycan cleavers (59). Deletion of *tagX* abolished *A. baumannii* and *A. baylyi* T6SS Hcp secretion and abrogated T6SS-killing of *E. coli* (59, 61). Despite this, TagX was found to be an accessory and not a core component, as T6SS TTC assembly and the ability to lyse *E. coli* were still observed in *tagX* deletion strains (61, 96). In addition, cells lacking *tagX* had reduced MC assembly (79). Purified TagX displayed peptidoglycan-degrading ability, consistent with its predicted role as an L,D-endopeptidase, while cellular fractionation confirmed its localization in the inner membrane with its C-terminus in the periplasm (59). TagX likely supports the T6SS by interacting with the MC, remodeling the bacterial cell wall, and allowing T6SS firing only at sites with legitimate target cells.

Though not present in T6SS loci, and not therefore a TAG, the transglycosylase MltE shows many similar phenotypes to TagX. MltE has been shown to be required for T6SS activity in EAEC (134). MltE is recruited to the MC to facilitate its assembly in the periplasm. The MC-MltE interaction augments MltE’s lytic transglycosylase ability, driving cell wall rearrangement. Deletion of *mltE* inhibits Hcp secretion and T6SS killing ability against *E. coli* K-12. These discoveries imply that some T6SS loci that lack a peptidoglycan rearranging gene may require endopeptidase machinery expressed from other locations in the genome, such as with the EAEC T6SS and MltE.

### TAGs with unknown functions

A large number of genes located in T6SS loci have been classified as Type VI-associated genes, but their mechanisms of function have either not been investigated or remain elusive. Examples of the former include the Bacteroidales-specific *tagA*, *tagB*, and *tagC* genes, while intriguing examples of the latter include *sfa3* (*sfnR2*) and *tagN*.

Bacteria of the Bacteroidales order are highly abundant gut residents who express the T6SS*^iii^* subtype in the mammalian GI tract (4, 20, 57, 135–137). T6SS*^iii^* loci have been classified into three genetic architectures (GA1-3) (67). All three GAs contain the core subcomplex machinery of TTC, BP, and MC, though the T6SS*^iii^* has a distinct MC (21). The accessory genes *tagA*, *tagB*, and *tagC* have unique distributions across the GAs. Within a GA, there is a high degree of sequence identity and positional layout of genes, with the greatest variability being for the effectors and immunity genes, which can vary even within a GA (67). In addition, GA1 and GA2 contain *tra* genes encoding horizontal gene transfer machinery as integrative and conjugative elements (ICE).

Bacteroidales strains bearing combinations of multiple GAs, especially GA1 and GA3, are commonly found in microbiome data sets. Recent data have shown that multiple GAs encoded by the same bacterium interact with each other, with GA1 inactivating GA3 secretion activity and becoming “dominant” (68, 138). This inhibitory effect is likely enhanced within the microbiome by GA1’s ability to transfer horizontally. In fact, within an individual’s microbiome, a single GA1 (with a unique repertoire of effector/immunity genes) is often found across multiple Bacteroidales and at the exclusion of other GA1 ICEs (4, 67, 139). In addition, GA2 and GA3 are rarely found together in a single bacterium, and when they are, one of them contains T6SS-inactivating mutations or mutations in an uncharacterized GA1-adjacent gene, *mhgA* (137). MhgA presumably has a role in controlling the co-localization of T6SS*^iii^* GAs within a single genome. Therefore, at both a population level and within a single bacterium, the T6SS*^iii^* GAs have complex interactions. With this established, the role of accessory genes in the Bacteroidales T6SS*^iii^* system may be significant.

Despite the importance of the T6SS*^iii^* in the gut community, little progress has been made in exploring the roles of *tagA*, *tagB*, and *tagC*. Thus far, only TagB has been experimentally investigated, with a *tagB* deletion mutant displaying a slight decrease in GA1 Hcp secretion (68). Understanding the role of uncharacterized TAGs, such as *tagA*, *tagB*, and *tagC*, in the dynamics of T6SS horizontal transfer, interactions between T6SS, and the regulation of T6SS activity is a significant area requiring further research.

Examples of TAGs with elusive functions are *S*fa3 and TagN. Discovered within the *Pseudomonas* T6SS-H3 system, the exact role of Sfa3 in T6SS activity has been difficult to determine (125). Located at the end of the T6SS-H3 locus, *sfa3* appears to have its own promoter (65). Its counterpart, *sfa2,* encoded in T6SS-H2, has been well-described as a Transcriptional Regulator. Sfa3 shares the same domains as Sfa2*,* except the GAF domain, and has been placed in the type 1C EBP class (65). Despite the sequence similarity to *sfa2* and a predicted interaction with RpoN, deletion of *sfa3* only yielded 10 differentially expressed genes in an RNA-seq analysis (65). The only T6SS-associated gene was an immunity gene, *tli5a*. However, H3-T6SS loci frequently contain *sfa3*, implying it may have a conserved, though unknown, role in T6SS activity.

TagN was first observed in *Burkholderia* T6SS-1 loci, where it was proposed to contain a peptidoglycan-binding domain to compensate for the “ancestral TssL’s” lack of a peptidoglycan-binding domain (140). Interestingly, *tagN* was found to be inhibited in *Acinetobacter* by the T6SS-repressing plasmid (59). The contribution of TagN to the T6SS has been difficult to determine. One group reported that *tagN’s* deletion slightly increased Hcp secretion, another that it had no effect, and another that secretion was slightly decreased (59, 61, 97). *A. baylyi tagN* deletion strains did have a moderately reduced competitive advantage and ability to lyse *E. coli* recipients, indicating some role in T6SS function (61). However, *A. baumannii* lacking *tagN* had no significant decrease in killing of *E. coli* recipients (97). Similarly, the effect of TagN on T6SS dynamics has been unclear, with one group observing reduced T6SS TTC dynamics with *tagN* deletion, and another reporting no changes (61, 96). Defining TagN’s role remains elusive. Speculatively, its activity may depend on particular recipients, a specific environment, or the state of the donor. However, *tagN’s* conserved presence in T6SS-encoding, clinically relevant pathogens, such as *Burkholderia* and *Acinetobacter*, argues for the need for further research regarding its mechanism.

### PAAR-like TAGs (TTC)

Though not a functional category within our framework, several genes in T6SS loci were preliminarily named TAGs which bear mentioning. These genes were named TAGs but lacked functional characterization. Later experimental work and/or improvements in domain predictions have revealed that these TAGs function as PAAR-like genes, rather than unique accessories. These proteins interact directly with VgrG at the tip of the TTC, or as chaperones for PAARs or effectors. We briefly highlight one example here, *tagAB* (BPS1504), as an interesting example of TAG research that has led to a better understanding of T6SS diversity.

*TagAB* was first discovered in the *Burkholderia* locus, where it was predicted to contain multiple pentapeptide repeats, have an N-terminal domain homologous to *tagA*, and have a C-terminus homologous to the adjacent gene, *tagB* (10)*. B. mallei* incubated with RAW macrophages revealed *tagAB* mRNA after 5 hours, showing its expression is induced during infections (70). *B. mallei* lacking *tagAB* had reduced Hcp secretion, lessened macrophage death, and reduced virulence in mice. Though the clinical significance of *tagAB* was clear, its functional role remained elusive.

A possible role for TagAB has since been proposed from research studying diverse methods bacteria employ in assembling the TTC. Previously, the TTC was thought to require both a VgrG and a PAAR, or PAAR-like domain (DUF4280, DUF4150), to fully assemble (23, 75, 141, 142). A growing body of literature has since found T6SS accessory proteins that have chaperone roles in effector recruitment to the TTC (45, 71, 143–146). In some cases, these accessories can compensate for T6SS that lack PAAR or PAAR-like proteins (147, 148). Recent work solved the structure of a PAAR-like protein from *Vibrio azureus*, finding a unique Pro-Iso-Pro-Tyr (PIPY) domain (DUF4150), rather than the canonical PAAR motif, as a sharpening structure for VgrG (72). Interestingly, ~80% of genomes assessed contained both a PIPY domain protein and a protein with an uncharacterized DUF2169 domain. The DUF2169 protein was found to be required for competition in *V. parahaemolyticus,* where binding to its cognate PIPY domain allowed VgrG secretion.

Intriguingly, TagAB contains a DUF2169 domain. It is predicted to belong to the “Multi-Domain DUF2169” gene synteny C (148). Though yet to be experimentally validated, TagAB may function as a chaperone for the TTC spike components in *Burkholderia*, rather than fitting in the six TAG functional categories.

## DISCUSSION

Through the course of evolutionary history, bacteria encounter new environments and competitors and are influenced themselves by stochastic processes such as genetic drift. The diversity of T6SS structural components, regulatory networks, effector/immunity pairs, and methods of deployment attests to the unique evolutionary trajectories diverse bacteria have experienced. We speculate that as bacteria develop nuances in the composition of their particular T6SS machinery, selection favors the acquisition of accessory factors that arise or are co-opted to sustain the fitness advantage provided by the T6SS. Many of these TAGs are not required for T6SS functionality in a binary sense. However, mutants of TAGs tend to display impairment in T6SS dynamics, control, architecture, and expression, indicating that they have roles in optimizing the function or activity of their cognate T6SS. Meanwhile, several genes termed T6SS-associated genes have been shown to be strictly necessary for T6SS activity as well.

Examples of TAGs have been found that impact T6SS activity at every step. Transcriptional Regulators, such as Sfa2, TagK, and VirAG, control the expression of T6SS gene clusters. T6SS firing is optimized by Target Recognition Factors, such as TagQRST, TslA, and TagM1, to mark contacts with target cells. These cell-cell sites become areas of T6SS assembly through the coordination of Secretion Regulators, such as TagE, TagH, and TagX. Proteins transcribed from core T6SS genes integrate with Structural Factors, including TagL, TsmK, and TagV, within the MC and BP to enhance the architecture of the T6SS. Finally, the T6SS TTC is tightly controlled from polymerization to contraction by Firing Coordinators, such as TsmA, TsmB, and TagJ, to minimize unproductive firing and maximize delivery of effector payload.

Although diverse TAGs have been discovered, many bacteria employing T6SS do not share TAGs. Rather, the functional categories of Firing Coordinators, Target Recognition Factors, Structural Factors, Transcriptional Regulators, and Secretion Regulators appear to be conserved aspects of T6SS functionality that interchangeable TAGs can facilitate. For example, TagQ and TslA are both Target Recognition Factors. TagQ is found in the *Pseudomonas* T6SS-H1 system, while TslA is present in the *Acinetobacter* T6SS. Though the T6SS systems are divergent, the functional need for target recognition is shared, and the functional need has been fulfilled with unique accessory proteins.

**Fig 2:**
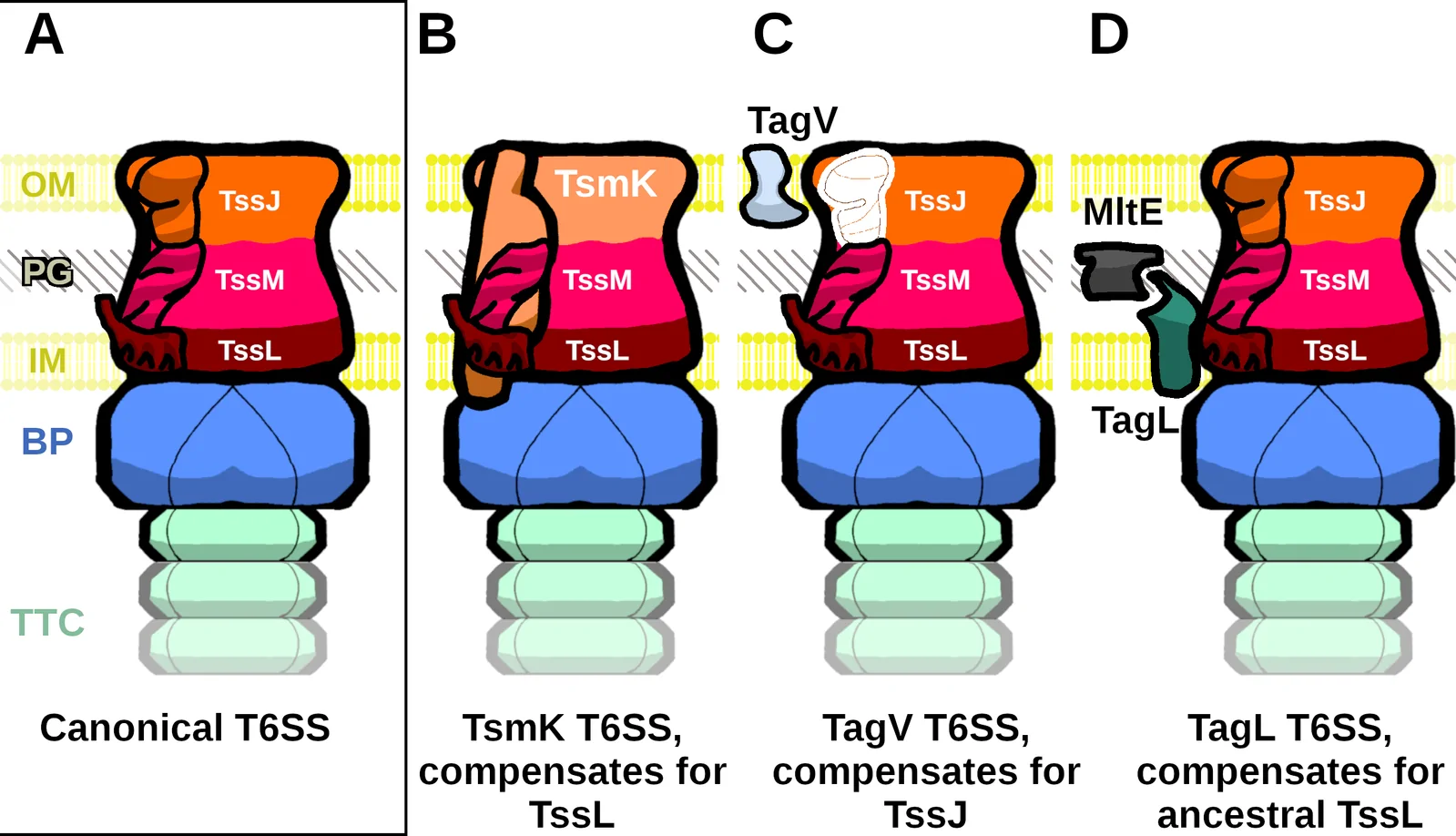
Compensatory roles of TAGs in the membrane complex. (**A**) Canonical T6SS membrane complex composed of TssJ (orange), TssM (pink), and “evolved/specialized” TssL (maroon). TssJML are shown as monomers. OM, outer membrane; PG, peptidoglycan; IM, inner membrane; BP, baseplate; TTC, tail-tube complex. (**B**) T6SS with TAG TsmK, which compensates for the lack of a TssL protein. TsmK in light orange. (**C**) T6SS with TagV, which can compensate for the lack of TssJ, although T6SS loci with TagV also encode TssJ. TagV in light blue. (**D**) T6SS with TagL. TagL, in combination with MltE, compensates for “ancestral” TssL that lack their own peptidoglycan-binding domains. TagL shown in dark teal, MltE shown in dark gray.

Another framework with which to classify TAGs is by compensatory or additive roles. During our literature review, we observed numerous situations where a variation in the core T6SS components was compensated by the presence of a TAG (Fig. 2). For example, *tsmK* is only found in T6SS loci that lack *tssJ*. T6SS loci with “ancestral” *tssL*, which lack peptidoglycan binding domains, also contain the peptidoglycan-binding *tagL*. TagV in *Serratia* is even able to facilitate productive Hcp secretion in a *tssJ* deletion background, which is inhibitory in other bacteria. Conversely, many TAGs provide additive roles that do not appear to compensate for known variations in the core genes. Examples of these TAGs include TslA, which recognizes OmpA in target cells to promote T6SS firing at cell-cell contacts, or TsmA, which binds TssA/TsmC at the distal end of the TTC to terminate excessive polymerization. This may serve as an alternative framework for classifying Unknown TAGs.

We wish to acknowledge the limitations of our definition of a TAG. We have constrained our discussion to genes within T6SS loci as facilitators of T6SS, though there are many examples of factors that impact the T6SS but whose genes are present elsewhere in the genome. Some examples include MltE, mentioned above as a transglycosylase similar to TagX; BsaN and BprC, transcriptional regulators of the *Burkholderia* T6SS; DsbA, which facilitates T6SS assembly in *Serratia* through disulfide bond formation of TssM; PhoPQ, a two-component system that activates T6SS in *Klebsiella* in addition to other virulence and antimicrobial peptide resistance genes; and ArgR, FNR, and Fur transcription factors in *Klebsiella* (70, 94, 112, 115, 131, 134, 149, 150). In addition, it is likely some TAGs have roles beyond their cognate T6SS. TagH, for example, has been shown in *V. cholerae* to inhibit *hlyA* transcription, even when its cognate T6SS lacks the canonical TagH activator TagE.

Finally, we suggest that our knowledge of TAGs is strongly biased by the model organisms that have been preferentially studied and sequenced in the field. We expect that as the diversity of known T6SS systems continues to increase, so too will TAGs. It is likely that TAGs will be discovered that do not adhere to the functional categories we present here. In addition, TAGs in the Unknown Function category require further research to discover their specific roles in T6SS activity. Because of the strong selective pressures on a bacterium to maintain a functional T6SS when in a competitive environment, we expect accessory genes that facilitate T6SS dynamics and regulation to experience similar selective pressures. Therefore, genes that are retained in T6SS loci but lack described functions, especially if their presence is well-distributed across evolutionarily-related taxa, likely are contributing to T6SS activity in significant ways. Many of these TAGs are expressed in clinically relevant bacteria, such as the gut-resident Bacteroidales (expressing *tagA*, *tagB*, *tagC*) or the pathogenic *Pseudomonas* (*sfa3*), *Burkholderia* (*tagN*), and *Acinetobacter* (*tagN*). Given the importance of the T6SS in bacterial fitness, the characterization of the roles of these genes in human-associated bacteria may yield insights relevant to health,

In conclusion, we hope that our framework formally organizing TAGs within functional roles will clarify and focus the field in the future and contribute to advancing our understanding of interbacterial competition across T6SS-encoding Gram-negative bacteria.
